# Over 20 Years of Machine Learning Applications on Dairy Farms: A Comprehensive Mapping Study

**DOI:** 10.3390/s22010052

**Published:** 2021-12-22

**Authors:** Philip Shine, Michael D. Murphy

**Affiliations:** Department of Process, Energy and Transport Engineering, Munster Technological University, T12 P928 Cork, Ireland; philip.shine@mtu.ie

**Keywords:** dairy, machine learning, artificial intelligence, precision agriculture, precision livestock farming

## Abstract

Machine learning applications are becoming more ubiquitous in dairy farming decision support applications in areas such as feeding, animal husbandry, healthcare, animal behavior, milking and resource management. Thus, the objective of this mapping study was to collate and assess studies published in journals and conference proceedings between 1999 and 2021, which applied machine learning algorithms to dairy farming-related problems to identify trends in the geographical origins of data, as well as the algorithms, features and evaluation metrics and methods used. This mapping study was carried out in line with PRISMA guidelines, with six pre-defined research questions (RQ) and a broad and unbiased search strategy that explored five databases. In total, 129 publications passed the pre-defined selection criteria, from which relevant data required to answer each RQ were extracted and analyzed. This study found that Europe (43% of studies) produced the largest number of publications (RQ1), while the largest number of articles were published in the Computers and Electronics in Agriculture journal (21%) (RQ2). The largest number of studies addressed problems related to the physiology and health of dairy cows (32%) (RQ3), while the most frequently employed feature data were derived from sensors (48%) (RQ4). The largest number of studies employed tree-based algorithms (54%) (RQ5), while RMSE (56%) (regression) and accuracy (77%) (classification) were the most frequently employed metrics used, and hold-out cross-validation (39%) was the most frequently employed evaluation method (RQ6). Since 2018, there has been more than a sevenfold increase in the number of studies that focused on the physiology and health of dairy cows, compared to almost a threefold increase in the overall number of publications, suggesting an increased focus on this subdomain. In addition, a fivefold increase in the number of publications that employed neural network algorithms was identified since 2018, in comparison to a threefold increase in the use of both tree-based algorithms and statistical regression algorithms, suggesting an increasing utilization of neural network-based algorithms.

## 1. Introduction

Animal agriculture is responsible for 14.5% of global anthropogenic greenhouse gases emissions, 20% of which are due to dairy production [1]. With a 22% increase in global milk production forecasted between 2018 and 2027 [2], it is essential that the dairy sector adequately addresses the significant challenges ahead to ensure the future sustainability of the global dairy industry. This is coupled with the rapid intensification of milk production systems that has taken place over the past 20 years. This increased intensification may be due to the principles associated with modern agricultural systems that define progress in terms of efficiency and productivity [3]. This has led to economies of scale throughout the dairy industry, with increasing herd sizes reducing fixed costs per unit output, coupled with an emphasis on maximizing output per hectare of farmland and per unit of input (e.g., concentrate feed). However, increased numbers of dairy livestock will naturally result in an increased workload for farmers, which may reduce income per hour worked or potentially reduce animal health and wellbeing, as farmers must care for increased numbers of livestock. Thus, dairy farmers are required to improve productivity (e.g., reduced production costs per litre of milk) without sacrificing milk production volumes, milk quality, or animal health and wellbeing. To achieve this, every aspect of the milk production cycle must be continuously monitored, evaluated and corrected to minimise the probability of undesirable farm events that can impact productivity and profitability.

The use of software and hardware technologies that support dairy farmers through the automation of on-farm decision making can help farmers facilitate increased herd sizes without added labor requirements. Machine learning algorithms and cognate methodologies can provide the necessary prediction accuracy to power these technologies through the ability to self-learn and improve over time when new data become available. Thus, there has also been an increased prevalence of machine learning algorithms employed throughout the dairy literature. As on-farm data collection technologies improve and become more commonplace in line with the rollout of the 5G network, the potential of these machine learning powered technologies will also increase [4]. Machine learning algorithms provide flexibility regarding data multicollinearity, input data distributions and missing data points while also having the ability to quantify interactions and non-linearities between features (i.e., independent variables) for regression and classification problems [5,6,7]. Machine learning algorithms include both supervised techniques (e.g., random forests), which require training data to find patterns, and unsupervised techniques (e.g., k-means clustering), which do not require training data to find patterns [8]. The ability of a machine learning model to provide accurate predictions and/or insights for on-farm decision making is directly related to the quality of input data used for model training. In addition, careful consideration must be given to ensure that a robust validation procedure is carried out (supervised learning), as model overfitting may result in a drastic overestimation of predictive capabilities. To realise the full potential of these algorithms, it is essential that best practice methodologies are identified and employed throughout the entire dairy research domain.

With the increased prevalence of machine learning algorithms throughout the dairy literature, the future direction of the research domain can be guided through the systematic mapping of the problems, features, algorithms and evaluation metrics and methods that have been employed to date. Recently, two studies have focused on reviewing the literature related to the applications of machine learning on dairy farms. Cockburn [9] summarised 97 studies related to dairy farm management, animal physiology, cow reproduction (animal husbandry), behavior analysis and feeding. The author followed a pre-defined search strategy and selection criteria, reviewed articles published between 2015 and 2020, and individually discussed each study within each subdomain. Study parameters, such as the dataset used, dependent variables, features and algorithms used, the prediction accuracy calculated and research design pitfalls, were discussed. Concurrently, Slob et al. [10] carried out a systematic mapping study of 38 primary studies published between 2010 and 2020 that applied machine learning for either disease detection in milk, forecasting milk production, or quantifying milk quality on dairy farms. Slob et al. [10] also followed a pre-defined search protocol and selection criteria to allow for reproducibility, as per the review guidelines outlined by Kitchenham et al. [11]. Similar to Cockburn [9], Slob et al.’s review focused on the problems addressed, the features and the machine learning algorithms used. However, Slob et al.’s mapping study contained a broader overview of the methodologies employed by highlighting common trends throughout the literature, as opposed to discussing the methods used by each individual study. Slob et al. also investigated the types of problems addressed (e.g., regression or classification), the evaluation parameters and validation approaches used, the most accurate algorithm per study and the challenges identified. However, Slob et al.’s mapping study did not incorporate studies from other subdomains within the dairy literature, such as animal health and wellbeing, farm management, feeding, animal reproduction and behavior analysis.

This systematic mapping study focused on collating and assessing studies published in journals and conference proceedings between 1999 and 2021, which applied machine learning algorithms to dairy farming related problems. Similar to Slob et al. [10], this mapping study followed guidelines outlined by Kitchenham et al. [11], whereby the research questions, search strategy and selection criteria were pre-defined. However, in contrast to Slob et al., (i) this study was not limited to publications solely within the cow milk subdomain; and (ii) this study did not exclude publications based on a quality score to ensure maximum coverage. Concurrently, in contrast to Cockburn [9]; (iii) this study was a mapping study, not a summary of the literature; and (iv) this study assessed the geographical location through categorising studies according to the continent of origin. In addition, in contrast to both Slob et al. and Cockburn, this study; (v) employed a much larger search period and far broader search strategy allowing for a greater number of publications to be identified and assessed; (vi) assessed publications over time in terms of research areas, algorithms and validation methods used to identify trends throughout the study period; (vii) quantified and presented research areas according to publication sources, as well as the feature categories used with different types of algorithms through the use of Sankey diagrams. Lastly; (viii) the top 10 most frequently used evaluation metrics for both classification and regression problems were assessed separately, as opposed to assessing these metrics together to allow for a more accurate representation of their respective popularities.

This article has four primary components; (1) a methodology section detailing said research questions, search strategy and selection criteria, in conjunction with data collection and data synthesis procedures; (2) a results section presenting findings that help answer each research question; (3) a discussion section highlighting common trends evident throughout the dairy literature, and (4) provides a concise conclusion to this review.

## 2. Methodology

This mapping study followed three primary stages, including: planning, conducting and reporting stages, as outlined by Kitchenham et al. [11]. In the planning stage, the research questions were defined, suitable databases identified and a robust search strategy was selected to identify the journal articles and conference papers (hereby referred to as publications) that could be used to answer the research questions. The databases were selected based on institutional access, their use in prior systematic literature reviews in the dairy research domain [9,10,12] and in conjunction with the ability or inability to easily carry out bulk downloads of publications. A heuristic approach was taken to identify the search string that provided a broad and unbiased search of the dairy literature without returning an unfeasible number of publications. During the conducting stage, the document search was carried out using the specifically defined search strings within each online database. The identified publications were filtered according to pre-determined selection criteria prior to analysis, whereby no quality assessment was performed in order to ensure maximum coverage. Relevant data required to answer each research question were then extracted from each publication and synthesised in the reporting stage via applicable charts, figures and tables.

### 2.1. Research Questions

The following research questions (RQ) were defined:

RQ1. What countries/regions are responsible for the largest number of publications?
RQ2. What journals and conference proceedings are research publications being published in?
RQ3. What problem areas are being addressed using machine learning in the dairy farming domain?
RQ4. What features are being employed to develop the machine learning models?
RQ5. What machine learning algorithms are being utilised to develop the models?
RQ6. Which evaluation metrics and methods are used?

### 2.2. Databases and Search Strategy

The literature search was carried out using five databases, Scopus, Science Direct, IEEE, Google Scholar and MDPI. These databases were selected as each allowed for the bulk downloading of publications (except for google scholar) while providing wide coverage of dairy-related research publications. Google Scholar returned a small number of publications; thus, bulk downloading was not required. A broad and unbiased search of the literature was undertaken to capture a wide range of publications from various areas within the dairy research domain [11] by using the search string “Dairy” AND (“machine learning” OR “artificial intelligence”). By default, each database’s search function also searched for the approximate search phrase “machine-learning”. This search string ensured that (1) preference was not given to any particular machine learning algorithm, and (2) a broad search of the literature was carried out without returning an unfeasible number of publications. Publications that contained the search string in either their *abstract*, *title* and/or *keywords* fields were identified using each database’s search function. However, Google Scholar did not allow for searches to be carried out on the publication’s *abstract* and *keywords* fields, so only the publication *title* field was used. The search strategy focused on identifying studies published between 1999 and 2021, whereby the last search was carried out on 9 June 2021. Initially, the search strategy aimed to identify studies published between 1990 and 2021; however, no studies were found prior to 1999. In total, 749 studies were identified between Scopus (n = 382), ScienceDirect (n = 109), IEEE (n = 189), Google Scholar (n = 45) and MDPI (n = 21) databases.

### 2.3. Selection Criteria

To filter only relevant publications required to answer the research questions (defined in Section 2.1), exclusion and inclusion criteria were determined, similar to Slob et al. [10]. To be included in the study, all exclusion criteria must be false, and all inclusion criteria must be true [11].

The exclusion criteria were:

1. The publication was not related to machine learning applied to dairy farming
2. The publication did not report empirical findings
3. The publication was not written in English
4. The publication was a duplicate study
5. There was no full text available
6. The publication was a review or survey study
7. The publication was published before 1999

The inclusion criteria were:

1. The publication features the development of machine learning models related to dairy farming
2. The publication is a primary study

### 2.4. Data Collection

**Figure 1:**
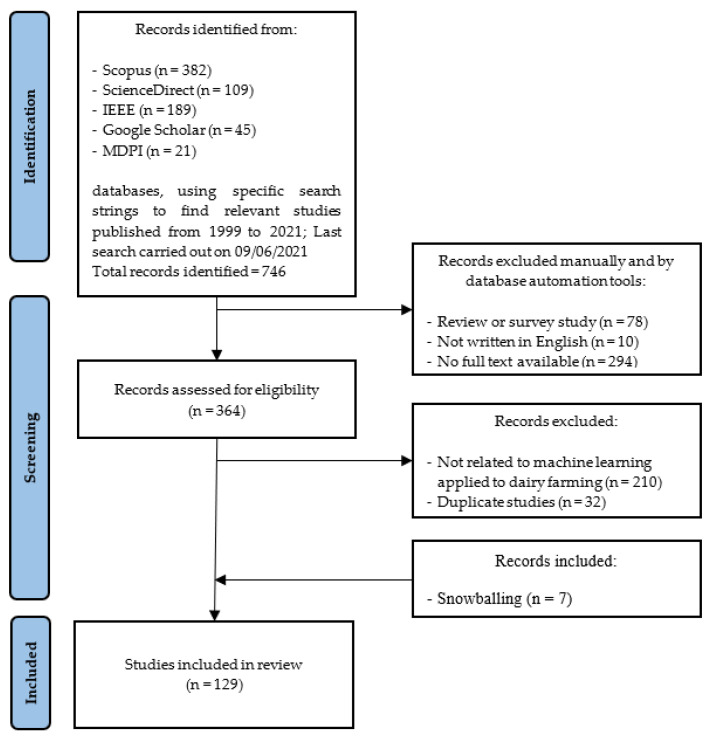
The flow of documents from identification to inclusion stage, in line with exclusion criteria.

Each publication identified by the search strategy outlined in Section 2.2 was analyzed relative to the exclusion and inclusion criteria (Section 2.3). The search strategy was carried out in line with PRISMA guidelines, as shown in Figure 1 [13]. The flow of documents from initial identification to the manuscript screening/filtering stage to the final subset of documents included in the mapping study is shown in Figure 1. The number of studies excluded due to each exclusion criterion is also highlighted at the screening stage. Of the 746 documents initially identified, 10 were not written in English, 78 were review/survey studies and 294 had no full text available for downloading from the database website. In addition, 210 publications were found to be outside the scope of developing machine learning models for dairy farming, while 32 documents were removed due to being duplicate studies. In addition, seven publications were included through snowballing, as employed by Slob et al. [10]. Cumulatively, 129 individual publications passed the selection criteria stage and were then included in the mapping study (Appendix E).

Relevant data were extracted from each of the 129 studies to respond to each of the six research questions. This was carried out by reading each publication and extracting the following information: (1) the year of publication, (2) publication source (name of the journal or conference proceedings), (3) whether the publication was a journal article or conference paper, (4) the country of origin (identified as country or countries where data collection took place), (5) the dependent variable or variables used, (6) the problem type (e.g., classification, regression or clustering), (7) the features employed, (8) the machine learning algorithms utilised, (9) the evaluation metrics used for synthesising model performance and (10) the validation technique used to quantify model performance.

### 2.5. Data Analysis

To ease with the synthesis of information, research categories, algorithm categories and feature categories were determined for each study. Categorisation was necessary to ensure each research question was addressed clearly and concisely. Firstly, each study was categorised according to its specific area of dairy research, whereby six categories were identified (RQ3) based on cognate review studies in the field: *physiology and health, animal husbandry, milk, feeding, management* and *behavior analysis*. Cockburn [9] employed physiology and health, animal husbandry, feeding, management and behavior analysis, while Slob et al. [10] assessed studies milk disease detection, quantifying milk production and milk quality. The range of dependent variables that were used to determine which of the six categories of dairy research each study related to is shown in Appendix A. Secondly, the machine learning algorithms used within each study were also categorised accordingly, whereby eight categories were identified (RQ5): *trees* (e.g., decision trees), *statistical regression* (e.g., multiple linear regression, ridge regression), *neural networks* (e.g., multi-layered perceptron, deep learning networks), *Bayes* (e.g., naïve Bayes, Bayesian-LASSO), *meta* (e.g., bagging, boosting), *rule-based* (e.g., Jrip, OneR), *clustering* (e.g., k-means, DBSCAN) and *other* (e.g., support vector machine, KNN). The full list of machine learning algorithms used and their corresponding category is shown in Appendix C. Additionally, the features used within each study were categorised accordingly, whereby 11 categories were identified (RQ4): *calving/pregnancy information, cow characteristics and clinical information, diet/feeding, farm characteristics and management, lactation information, meteorological conditions, milk characteristics, milking parameters, sensors, soil characteristics* and *other*. The full list of features used and their corresponding categories are shown in Appendix B. Lastly, the categorisation of journals and conference proceedings was also carried out to help improve data synthesis. The *other journal* category represented journals that had less than four published articles included in this study, while all conference papers were included in a *conference paper* category (RQ2). The full lists of journals and conferences proceedings are shown in Appendix D.

Categorisation was straightforward when a publication focused on only one dependent variable. However, 13 publications focused on the prediction of multiple dependent variables. In these cases, the problem type, algorithms employed and features used were recorded for each dependent variable. Each dependent variable was categorised according to its specific area of dairy research. When a publication focused on the prediction of multiple dependent variables, each attributable to a different area of dairy research, each dependent variable was treated as a separate study. Otherwise, information would be excluded when; assessing the frequency of studies published in different research areas over time (RQ3), investigating the geographical locations attributed to different research areas (RQ1) and when evaluating the research problem type and popular journals and conference proceedings associated with different areas of dairy research (RQ2).

When a publication focused on the prediction of multiple dependent variables in the same dairy research area but utilising different features, each study involving unique sets of features were treated as a separate study. However, this was only applicable when addressing RQ4, whereby the features employed in different research areas in conjunction with the machine learning algorithms used was investigated. Otherwise, information related to the features used within each research area would be excluded.

Three studies involved the collection of data in more than one country/region. In such instances, each country was treated as though it had independently carried out the study. This was applicable when assessing the geographical distribution of the publications (RQ1). Assessing the geographical locations of publications was carried out on an individual publication basis, irrespective of the number of dependent variables. Likewise, assessing the algorithms used (RQ5), validation methods and model performance metrics used (RQ6) throughout the literature were carried out on an individual publication basis, as these were found to be consistent throughout each publication irrespective of the number of dependent variables.

## 3. Results

### 3.1. Geographical Distribution

**Figure 2:**
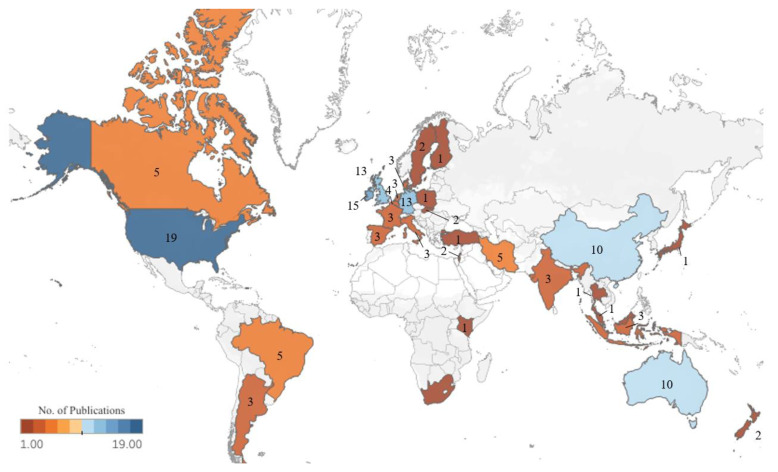
Geographical distribution of research studies (n = 139).

**Figure 3:**
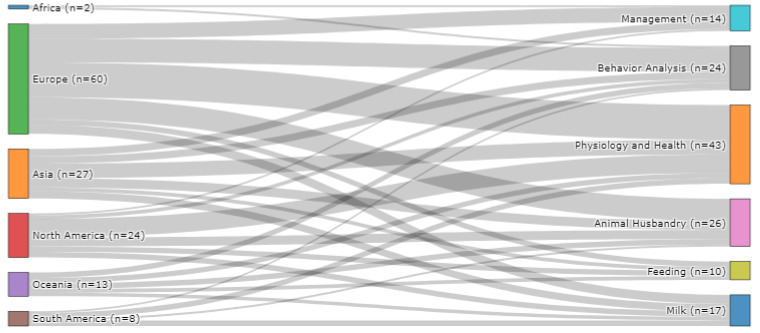
The flow of studies from geographical location to research categories (n = 134).

The geographical distribution of the publications included in this study is shown in Figure 2. The geographical location was determined by the origin of the data used for model development. In total, 30 countries contributed data to machine learning in the dairy farming research domain. Data originated from one single country for 126 of the studies, with the remaining three studies having cross-border collaboration. These included collaborations between: (1) the United Kingdom, Italy, Sweden and Finland; (2) Australia, Canada, Denmark and Ireland; and (3) Belgium, Canada, Ireland, Denmark and Germany. In relation to RQ1, the largest number of studies utilised data originating from the United States (n = 19), followed by Ireland (n = 15), Germany (n = 13) and the United Kingdom (n = 13), and Australia (n = 10) and China (n = 10). The remaining 24 countries contributed data to five or fewer research publications. However, from a continental perspective, Europe (n = 60) was by far the largest contributor of data, followed by North America (n = 24) and Asia (n = 27), Oceania (n = 13), South America (n = 8) and Africa (n = 2). Data originating from Europe were used in studies focusing on the physiology and health of dairy cattle (n = 19), analysing animal behavior (n = 13), animal husbandry (n = 12), farm management (n = 8), milk (n = 5) and feeding (n = 3), as shown in Figure 3. Applying machine learning algorithms to assess the physiology and health of dairy cattle was also the most popular research category for the North America (n = 10) and Asia (n = 8) continents and joint most popular category in Oceania (n = 3) and South America (n = 3).

### 3.2. Publications Timeline

**Figure 4:**
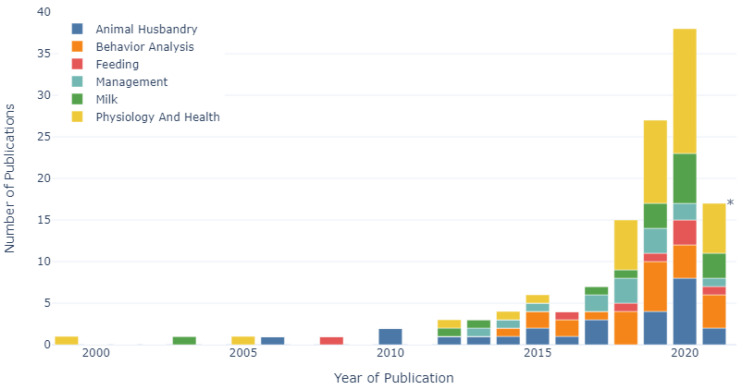
Number of publications per year labelled according to research category (n = 131). * Data collected up to June 2021.

The number of research studies published per year from 1999 to 2021, categorised according to each research area, is shown in Figure 4. Prior to 2018, the animal husbandry category was the largest research area representing 35% of all publications in that period, followed by behavior analysis (19%), management (15%) and physiology and health (15%). A significant increase in the number of publications occurred in 2018, whereby a total of 15 journal articles and conference papers were published, representing a 114% increase compared to 2017. This trend continued in 2019 and 2020, whereby year-on-year increases of 80% and 41% were recorded, respectively. This resulted in 74% of the publications included in this mapping study being published after 2017, representing a threefold increase. On average, between 2018 and 2021, the physiology and health research category was the largest research area (38%) (up from 15% between 1999 and 2017), followed by research related to behavior analysis (19%) and animal husbandry (14%). The physiology and health research category represented the largest research area in each year between 2018 and 2021, representing 40%, 37%, 39% and 35% of publications, respectively. Behavior analysis was the second-largest research category in 2018 (27%), 2019 (22%) and the first five months of 2021 (24%), while animal husbandry was the second-largest research category in 2020 (21%).

### 3.3. Publications Breakdown

The following section has two primary components: the first component provides a breakdown of the type of problems addressed in relation to the source journals that published the research studies and the areas of research that machine learning has been applied to throughout the literature. The second component provides a breakdown of each research area in relation to the features considered for model development and machine learning algorithms employed.

#### 3.3.1. Problem Type, Journals/Conferences and Research Area

**Figure 5:**
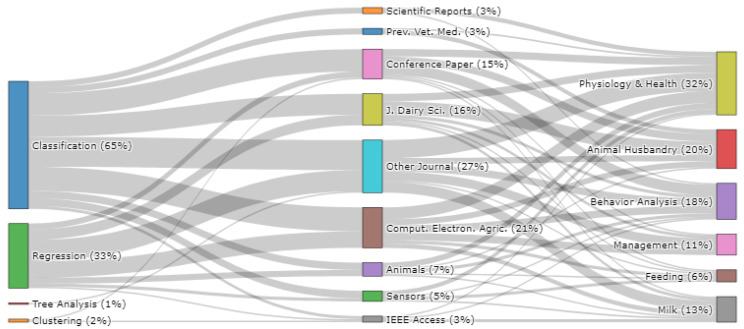
The flow of studies from problem type to publication source to research categories (n = 131).

The flow of research studies from the type of problem addressed, to the publication destination, to the area of research carried out is shown in Figure 5. Overall, 65% of the research studies focused on addressing classification problems, 33% addressed regression problems, while 2% and 1% focused on clustering and tree analysis problems, respectively. In relation to RQ2, the Computers and Electronics in Agriculture journal was responsible for publishing the largest number of research studies (21%), followed by the Journal of Dairy Science (16%). In addition, 27% of all research studies were published in *other journals* (Appendix D), whereby each journal was responsible for publishing less than four research articles included in this study, while 15% of all publications (20 conference papers) were published in 18 different conference proceedings. Concurrent with Section 3.2, and in relation to RQ3, the majority of studies focused on physiology and health research (32%), followed by animal husbandry (20%), behavior analysis (18%), milk (13%), management (11%) and feeding (6%). No clear trend or bias was found between the types of problems addressed and the publication sources, whereby the most popular destination for both classification and regression problems was the *other journals* category, followed by the Computers and Electronics in Agriculture journal. Regarding the destination of each publication in relation to the research area, the largest number of research publications published in *other journals* and the Computers and Electronics in Agriculture journal focused on physiology and health applications (n = 12 and n = 8, respectively). However, this varied from articles published in the Journal of Dairy Science, where the largest number of research articles focused on animal husbandry applications (n = 9).

#### 3.3.2. Research Area, Features and Algorithms Used

**Figure 6:**
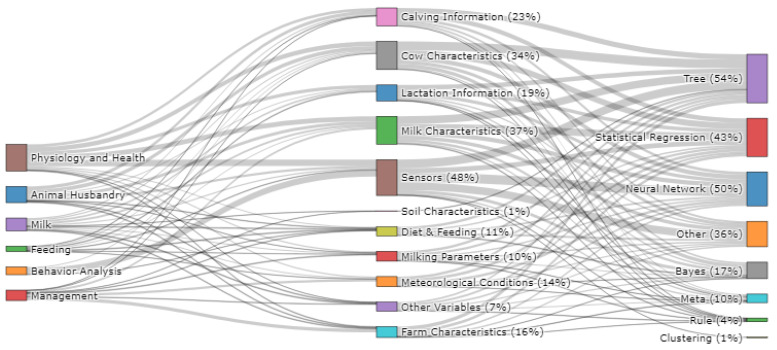
Flow of studies from research area to features categories to algorithm categories (n = 134).

The flow of research studies from a research category to the category of features considered to the category of machine learning algorithms is shown in Figure 6. Overall, 48% of research studies utilised sensor data for model development (RQ4), predominantly for physiology and health (n = 24) and behavior analysis (n = 24) applications. Accelerometer (n = 27), image (n = 7) and pedometer (n = 6) data were the three most frequently employed types of data collected by sensors, as shown in Appendix C. Sensor data were most frequently employed as feature data when developing artificial neural network models (n = 35), tree-based models (n = 32) and *other* model types (n = 31), whereby *other* models included the application support vector machine and k-nearest neighbor algorithms (full list shown in Appendix C). In addition, cow characteristics (34%), milk characteristics (37%), calving information (23%) and lactation information (19%) were also commonly employed as feature data followed by meteorological data (14%), diet and feeding (10%), farm characteristics (16%), milking parameters (10%), soil characteristics (1%) and *other variables* (7%). Regarding the algorithms employed (RQ5), tree-based algorithms were employed in the largest number of studies (54%), followed by neural network algorithms (50%), statistical regression-based algorithms (43%), *other* model types (37%), Bayes algorithms (17%), meta (10%), rule (4%) and clustering (1%). A full breakdown of the specific algorithms employed within each algorithm category is shown in Appendix C, in conjunction with the number of studies that each algorithm was employed.

**Figure 7:**
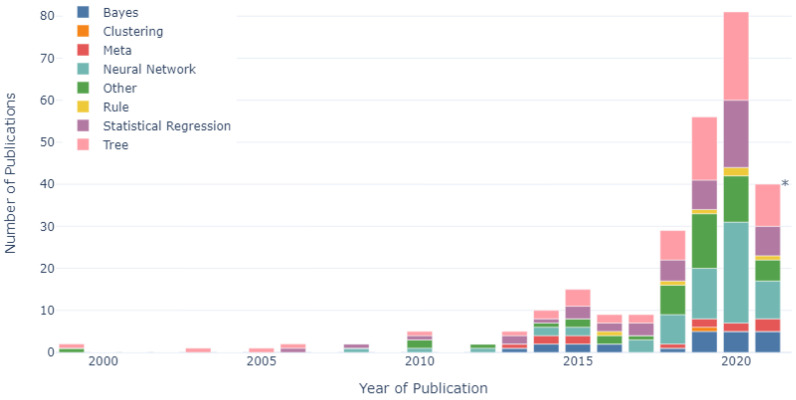
Number of publications per year labelled according to algorithm category (n = 269). * Data collected up to June 2021.

The number of research studies published per year from 1999 to 2021, categorised according to each algorithm method, is shown in Figure 7. Prior to 2018, tree-based algorithms were the most frequently employed algorithm category (employed in 25% of all publications), followed by statistical regression-based algorithms (22%). This trend continued in the period between 2018 and 2021, whereby the percentage of publications that employed tree-based algorithms increased to 26%. However, the percentage of publications that employed statistical regression algorithms reduced to 17%, while the percentage of publications that employed neural network-based algorithms increased to 25% during the 2018 and 2021 period (up from 16% between 1999 and 2017). This equated to a fivefold (5.2), or a 420% increase in the number of publications that employed neural network algorithms since 2018, in comparison to a threefold (3.3) increase in the number of publications that employed tree-based algorithms and statistical regression algorithms (2.5).

### 3.4. Evaluation Metrics Used

In relation to RQ6, the ten most frequently used evaluation metrics for assessing regression and classification problems are shown in Table 1, in conjunction with the percentage of studies each metric was used in. For studies that focused on regression problems (n = 41), root mean squared error (RMSE) was the most frequently employed metric, whereby it was used in 56% of studies, followed by the coefficient of determination (R^2^) used in 46% of studies, correlation coefficient (r) (27%), mean absolute error (MAE) (24%), concordance correlation coefficient (CCC) (17%), mean absolute percentage error (MAPE) (15%), mean squared error (MSE) (15%), relative prediction error (RPE) (15%), mean percentage error (MPE) (10%) and mean squared percentage error (MSPE) (7%). In relation to studies that focused on classification problems (n = 85), classification accuracy was the most commonly employed evaluation metric (77%), followed by recall (66%), specificity (49%), positive predictive value (PPV) (48%), F_1_ Score (27%), the area under the ROC curve (AUC) (26%), negative predictive value (NPV) (15%), Cohen’s K (12%), false positive (FP) (9%) and false negative (FN) (6%).

### 3.5. Validation Methods

In relation to RQ6, six evaluation methods were identified throughout the 127 studies that addressed classification, regression and clustering (n = 1) problems: hold-out cross-validation (n = 49), leave-out-one-animal (LOOA) (n = 4), leave-one-out cross-validation (LOOCV) (n = 3), nested cross-validation (Nested CV) (n = 7), Train/Validation/Test (n = 17) and k-fold cross-validation (n = 30), as shown in Table 2. The k-fold cross-validation method was employed with a mean *k* value of 10, the hold-out method was employed with 71% of data used for training and 29% of data used for a test dataset, while the train/validation/test method used 65%, 17% and 18% of data for training, validation and testing, respectively. In 21 research studies, these evaluation methods were repeatedly carried out to reduce the probability of biased results associated with a single hold-out, train/validation/test or k-fold CV split. The number of studies that repeatedly carried out each particular evaluation method is highlighted in brackets. On average, the hold-out method was repeated 38 times, the train/validation/test method was repeated 10 times and k-fold cross-validation was repeated 14 times. In addition, 16 research studies employed a combination of two evaluation methods to further separate training and testing stages, particularly important for when tuning hyper-parameters. For example, 15 studies employed k-fold CV for model training to select features and/or hyper-parameters and calculated prediction accuracy on separate test data using hold-out cross-validation. One study employed two different evaluation methods for two different dependent variables.

**Figure 8:**
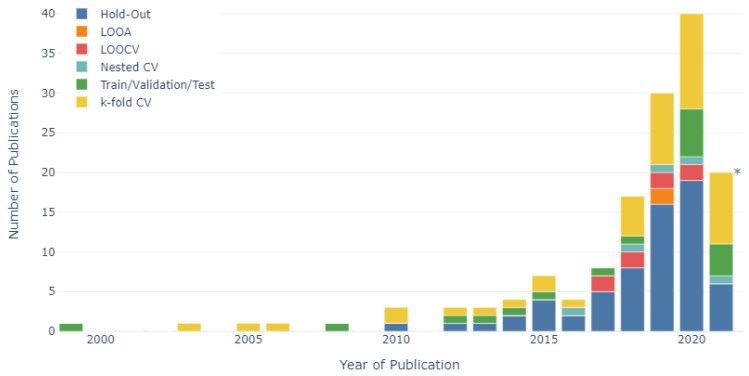
Number of publications per year labelled according to validation method used (n = 127). * Data collected up to June, 2021.

The number of research studies published per year from 1999 to 2021, categorised according to each validation method, is shown in Figure 8. Prior to 2018, the hold-out method was the most frequently employed validation method (employed in 43% of all publications), followed by k-fold cross-validation (30%) and train/validation/test validation (19%). This trend continued throughout the 2018 to 2021 period, whereby the percentage of publications that employed the hold-out method increased slightly to 46%, as did the use of k-fold cross-validation (33%). However, this period also saw a reduction in the percentage of publications that employed the train/validation/test validation (10%). The hold-out cross-validation method was the most frequently employed method each year between 2014 and 2020, while the k-fold cross-validation method was the most frequently used method (45%) in the first five months of 2021. In 2019 and 2020, the use of the hold-out method increased by 100% and 19%, year-on-year, respectively, while the use of k-fold cross-validation increased by 80% and 33%, year-on-year, respectively.

## 4. Discussion Overview

This study represents the largest and broadest systematic mapping review to date, focusing on published literature related to the application of machine learning algorithms in the dairy research domain. In total, 129 publications were included and assessed, made possible due to a combination of broad search terms and an increased search period spanning over 21 years. However, it is still plausible that additional publications that focused on the application of machine learning algorithms on dairy farms were not captured by the search strategy employed. The search strategy involved five databases chosen to provide wide coverage of dairy-related research while allowing for the bulk downloading of publications. It is likely that some publications located in other databases were not included. Snowballing was carried out to help reduce the number of publications not included. However, the largest barrier to including publications in this study was the availability of a full text from the Scopus database. This was due to restrictions on the publisher’s side, which accounted for 93% of the total number of excluded publications.

Throughout the 129 publications included in this mapping study, a considerably wide range of dependent variables (n = 66), features (n = 251) and algorithms (n = 90) were employed in 35 journals and 18 conference proceedings. It was, therefore, necessary to categorise dependent variables, features, algorithms and journal articles and conference papers accordingly to ensure findings could be easily digested and each research question could be adequately addressed. Categorisation was based on the experience of the authors while considering the categorisation approaches employed in cognate studies. This included the categorisation of: (1) each dependent variable into one of six research categories, (2) each feature into 1 of 11 feature categories, (3) each algorithm into one of eight algorithm categories and (4) journals that published four or fewer articles included in this study into the *other* journals category, and all conference papers into a separate *Conference Paper* category. For full transparency, the full lists of dependent variables, features and algorithms employed and their respective categories, as well as the journal/conference proceedings, are presented in Appendix A, Appendix B, Appendix C and Appendix D respectively.

All neural network-based models, including multilayer perceptron networks, convolutional neural networks and long-short term memory networks, were included in the *Neural Network* category to minimise the over-categorisation of algorithms. The number of studies that employed each neural network-based algorithm can be found in Appendix C.

The research categories, algorithm categories and validation methods employed per year were assessed between 1999 and 2021 to allow for trends in research areas and methodologies to be identified over time. Firstly, regarding the research categories, the largest number of publications prior to 2018 were related to animal husbandry (35%). However, since 2018, the largest number of publications have been related to physiology and health (38%), with the percentage of publications focusing on animal husbandry research reducing to 14%. This suggested a trend throughout this research domain, with studies moving away from animal husbandry-related problems to focus on improving the physiology and health of dairy cows. The number of studies that focused on the physiology and health of dairy cows has increased seven-fold since 2018. Concurrently, the smallest number of publications both prior to 2018 (6%) and after 2018 (6%) were related to feeding, suggesting an opportunity for future research to be carried out in this largely unexplored subdomain. Secondly, in relation to the types of algorithms employed, tree-based algorithms were the most frequently employed algorithm category, being used in 25% and 26% of studies prior to 2018 and since 2018, respectively. However, the use of statistical regression-based algorithms reduced from 22% to 17%, before and after 2018, respectively, while at the same time, the use of neural network-based algorithms increased from 16% to 25%. This suggested a move away from statistical regression-based algorithms towards the utilisation of neural network-based algorithms. Lastly, regarding the validation methods employed, both prior to 2018 and after 2018, hold-out cross-validation was the most frequently employed validation method, being used in 43% and 46%, respectively. In addition, the use of k-fold cross-validation also increased from 30% to 33% during these periods. However, the percentage of studies that used the train/validation/test validation method reduced from 19% to 10% before and after 2018, respectively, suggesting a trend away from the train/validation/test method towards hold-out and k-fold cross-validation.

This mapping study was carried out in line with PRISMA guidelines, with six pre-defined research questions outlined in Section 2.1. The search strategy produced results that adequately addressed each research question. In relation to RQ1, the country responsible for the greatest number of publications was the USA (n = 19); however, when the geographical location of studies was assessed on a continent basis, Europe was by far the greatest region, producing 60 publications. Regarding RQ2, the greatest number of publications was published in the Computers and Electronics in Agriculture journal (21%), followed by the Journal of Dairy Science (16%). Additionally, 35 publications (27%) were published across 28 other journals that each published less than four papers included in this study, while the 20 conference papers were published in 18 different conference proceedings. RQ3 focused on determining what research areas were being addressed in the dairy research domain using machine learning methodologies, where results showed that the greatest number of studies addressed problems focused on the physiology and health of dairy cows (32%). In relation to RQ4, the most frequently employed feature data throughout the literature were derived from sensor data (48%), with 27 studies employing accelerometer data. Additionally, RQ5 focused on identifying the most frequently utilised machine learning algorithms used throughout the dairy literature. The greatest number of studies employed tree-based algorithms (54%), followed by neural network-based algorithms (50%). Lastly, RQ6 focused on identifying the evaluation metrics and methods employed throughout the dairy literature. Assessing the literature showed that RMSE (56%) and R2 (46%) were the most frequently employed metrics used for regression problems, while accuracy (77%) and recall (66%) were the most frequently employed metrics used for classification problems. In addition, hold-out cross-validation was the most frequently employed evaluation method throughout the literature.

## 5. Conclusions

The results show that there has been a considerable increase in the prevalence of published literature applying machine learning algorithms to help solve problems on dairy farms, with 74% of the publications included in this study published since 2018. Europe was responsible for the production of data utilised in 45% of the research studies assessed, highlighting the need for an increase in research studies in other regions, in particular Africa, Oceania and South America. In addition, 32% of the studies included in this review applied machine learning to problems related to the physiology and health of dairy cows, with a seven-fold increase in publications in this area occurring since 2018. Concurrently, this study has also highlighted a reduction in the percentage of studies that used statistical regression algorithms coupled with an increased percentage of studies that used neural network-based algorithms since 2018, when compared with the 1999 to 2017 period. As machine learning algorithms are more-frequently applied to problems in the dairy domain, it is important that best practice guidelines are followed to ensure their potential impact is realised. This mapping study may be used as the basis for future research in the dairy domain to identify studies that may have focused on a similar problem, whereby an identical, similar or improved methodology may be suitable.

## Figures and Tables

**Table 1:** Percentage of studies using each evaluation metric for classification and regression problems.

**Regression (n = 41)**										
	**RMSE**	**R^2^**	**r**	**MAE**	**CCC**	**MAPE**	**MSE**	**RPE**	**MPE**	**MSPE**
% of studies	56%	46%	27%	24%	17%	15%	15%	15%	10%	7%
**Classification (n = 85)**										
	**Accuracy**	**Recall**	**Specificity**	**PPV**	**F_1_ Score**	**AUC**	**NPV**	**Cohen’s K**	**FP**	**FN**
% of studies	77%	66%	49%	48%	27%	26%	15%	12%	9%	6%

RMSE = root mean squared error; R^2^ = coefficient of determination; MAE = mean absolute error; MSE = mean square error; CCC = concordance correlation coefficient; MAPE = mean absolute percentage error; RPE = relative prediction error; MPE = mean percentage error; MSPE = mean square percentage error; PPV = positive predictive value; AUC = area under the ROC curve; NPV = negative predictive value; FP = false positive; FN = false negative.

**Table 2:** Number of studies employing each evaluation method(s) (n = 127).

Evaluation Method ^a^	Hold-Out	LOOA	LOOCV	Nested CV	Train/Validation/Test	k-Fold CV
Hold-Out	49 (5) ^b^	-	-	-	-	-
LOOA	-	4	-	-	-	-
LOOCV	1	-	3	-	-	-
Nested CV	-	-	-	7	-	-
Train/Validation/Test	-	-	-	-	17 (1)	-
k-fold CV	15 (4)	-	-	-	1 ^c^	30 (11)

LOOA = leave-out-one-animal; LOOCV = leave-one-out cross-validation; Nested CV = nested cross-validation; k-fold CV = k-fold cross-validation. ^a^ Values along the diagonal refer to the number of studies that used that particular evaluation method. Values not along the diagonal refer to the number of studies that used a combination of evaluation methods corresponding to the value’s vertical and horizontal position. ^b^ Bracketed values represent the number of studies where that particular evaluation method was carried out repeatedly (i.e., more than once). ^c^ One study employed two different evaluation methods for two different dependent variables.

## Data Availability

Data were compiled from five databases: Scopus (https://www.scopus.com/, accessed on 9 June 2021), Science Direct (https://www.sciencedirect.com/, accessed on 9 June 2021), IEEE (https://ieeexplore.ieee.org/, accessed on 9 June 2021), Google Scholar (https://scholar.google.com/, accessed on 9 June 2021) and MDPI (https://www.mdpi.com/, accessed on 9 June 2021).

## Appendix A

The specific dependent variables used per research category are shown in Table A1, with the number of studies that used each dependent variable presented in brackets next to each variable. The total number of dependent variables per category is also presented.

**Table A1:** Specific dependent variables used per research category.

Category	Dependent Variables (Number of Studies)	n
Animal Husbandry	Estrus Detection (7), Pregnancy Status (6), Calving Prediction (3), Cow Survival (2), Abortion Incidence (1), Calving Difficulty (1), Conception Performance (1), Conception Probability (1), Conception Success (1), Conception Rate (1), First-Service Conception Rate (1), Genomic Evaluation (1), Service Rates (1), Submission Rate (1)	14
Behavior Analysis	Cow Activity (17), Cow Detection (3), Cow Identification (2), Jaw Movements (1), Sleep Stages (1)	6
Feeding	Dry Matter Intake (2), Concentrate Feed Intake (1), Diet Energy Digestion (1), Feeding Behavior (1), Insufficient Herbage Allowance (1), Residual Feed Intake (1), Volatile Fatty Acids (1)	7
Management	Electricity Use (6), Energy Output (3), Methane Emissions (2), Water Use (2), Diesel Use (1), Faecal Nitrogen (1), Faeces Output (1), Herbage Production (1), Manure Temperature (1), Nutrient Concentration (1), Urinary Nitrogen (1), Urine Output (1)	12
Milk	Milk Production (6), Milk Adulteration (4), Milk Quality Parameters (2), Fat EBV (1), Milk Bacterial Index (1), Milk EBV (1), Milk Metabolites (1), Milk Parameters (1), Outlier Lactations (1)	9
Physiology and Health	Mastitis Detection (11), Lameness Detection (10), Body Condition Score (7), Heat Stress (4), Bodyweight (2), Metabolic Status (2), Animal Dimensions (1), Digital Dermatitis (1), Ketosis Detection (1), Milk Productivity (1), Noxious Events (1), Respiration Rate (1), Rumen and Blood Metabolites (1), Skin Temperature (1), Teat Cleanliness (1), Tuberculosis Status (1), Vaginal Temperature (1)	17

## Appendix B

The specific features used per feature category are shown in Table A2, with the number of studies that utilised each feature presented in brackets next to each feature. The total number of features per category is also shown.

**Table A2:** Specific features used per feature category.

Independent Variable Category	Features (Number of Studies)	n
Calving/Pregnancy Information	Parity (24), Calving Interval (5), Previous Calving (2), AI Season (1), AI Stage (1), Calf Sex (1), Calving Age (1), Calving Month (1), Conception Rate (1), Days Since Previous AI (1), Displaced Abomasum (1), Duration of The Voluntary Waiting Period (1), Fertility EBI (1), Length of Pregnancy (1), Month of Insemination (1), Negative Energy Balance (1), No. of Heifers Calved (1), No. of Lactating Cows (1), No. of Previous Inseminations (1), Number of Cows In The Maternity Pen (1), Pregnancy Status (1), Pregnancy Stage (1), Previous Abortion (1), Previous Year’s Conception Rate (1), Reproduction Performance (1), Strategy For Using A Clean-Up Bull (1), Temperature For Thawing Semen (1)	27
Cow Characteristics and Clinical Information	Bodyweight (11), Age (5), Breed (5), Genetics (5), BCS (4), Heart Rate (4), Body Temperature (3), Mastitis Detected (3), Phenotype Data (3), Breeding Values (2), Core Rumen Microbiome (2), Ketosis (2), Survival (2), Veterinary Treatments (2), Accumulated Number of Mastitis Cases (1), Back Fat Thickness (1), Bacteriological analysis (1), Blood Oxygen Saturation (1), Body Mass (1), Bodyweight Leg Distribution (1), Breathing Rate (1), Clinical Case Ratio (1), Clinical Mastitis (1), Core Temperature (1), Cytometric Fingerprint (1), EBV (1), Estrus Detected (1), Health (1), Lameness (1), Longevity (1), Medical Conditions (1), Medication (1), Metritis (1), Microrna Gene Expression Data (1), Percentage of Cows With Low BCSs (1), Previous BCS (1), Proportion of Hf Genes In Cow Genotype (1), Retained Placenta (1), Reticulorumen Temperature (1), Ruminal pH (1), Sire and Dam Fat EBV (1), Sire And Dam Milk EBV (1), Teat Sanitation (1), The Frequency of Hoof Trimming Maintenance (1), Udder Depth (1)	45
Diet/Feeding	Diet Composition (3), Feed Intake (2), Programmed Concentrate Feed (2), Concentrate Feed (1), DMI (1), Drinking Duration (1), Eating Duration (1), Feed Bin Visits (1), Forage Species (1), Mean Duration of Trough Visits (1), Nutrient Management (1), Pasture Composition (1), Roughage Feed (1), Rumination Time (1), TMR Composition (1), Total Feed Intake (1), Vitamins (1), Water Bin Visits (1), Water Intake (1)	19
Farm Characteristics and Management	Herd Size (9), No. of Parlour Units (7), Frequency of Hot Wash (6), Hot Water Tank Volume (6), Milk Cooling System (4), Milk Tank Volume (4), No. of Air Compressors (4), No. of Scrapers (3), Electricity Energy (2), Field Troughs (2), Flow Rate (2), Fossil Fuel Energy (2), Housing (2), Milk Pre-Cooling (2), Parlour Washing (2), Rainwater Collection (2), Air Conditioning (1), Bunk Space Per Cow (1), Facilities (1), Fan (1), Farm Management (1), Feed Energy (1), Feed Supply Energy (1), Fuel Energy (1), Grazing Management (1), Hectares (1), Herd Management (1), Human Labour Energy (1), Indoor Temperature (1), Labour (1), Labour Energy (1), Lime Management (1), Logistics Pickup (1), Machinery Energy (1), Manure Depth (1), Mechanised Feeding (1), No. of Scrapers (1), Pasture Management (1), Room Temperature (1), Stocking Rate (1), Tank Cleaning (1), Tank Level (1), Type of Bedding In The Dry Cow Pen (1), Type of Cow Restraint System (1), Water Energy (1)	45
Lactation Information	DIM (19), Complete Lactation (1), Dry Period (1), Dry Period Cure Rate (1), Dry Period Length (1), Early Lactation (1), Freshening Date (1), Lactation Stage (1), Week of Lactation (1)	9
Meteorological Conditions	Ambient Temperature (15), Relative Humidity (11), Rainfall (6), Wind Speed (6), Wind Direction (4), Dewpoint Temperature (3), Solar Radiation (3), Wet Bulb Temperature (3), Dry Bulb Temperature (2), Air Pressure (1), Air Temperature (1), Black Globe Temperature (1), Degree Days Below 15 C (1)	13
Milk Characteristics	Milk Yield (34), Milk Fat (20), Milk Protein (19), Milk Lactose (10), SCC (10), Milk Conductivity (5), Milk MIR Spectral Data (5), Milk Temperature (5), Milk Fatty Acids (3), Milk Flow (3), 305 Day MY Equivalent (2), Milk Density (2), Milk Ph (2), Milk SNF (2), 305 Day FPCM Equivalent (1), Blood In Milk (1), Fat Corrected Milk (1), Max Fat/Protein Ratio of Previous Lactation (1), Metabolite Data (1), Milk Acetone (1), Milk Casein (1), Milk Fever (1), Milk Freezing Point (1), Milk Genetics (1), Milk Infrared Spectroscopy Data (1), Milk Mineral Content (1), Milk Persistency (1), Milk Urea (1), Non-Esterified Fatty Acids (1), Saturated Fatty Acids (1), Single Nucleotide Polymorphism Markers (1), Specific Gravity (1), Unsaturated Fatty Acids (1), Urea (1)	34
Milking Parameters	Milking Frequency (4), No. of Vacuum Pumps (3), Milking Duration (2), Milking Time (2), Peak Milk Flow (2), Cups Kicked off During Milking (Yes/No) (1), Expected Milk Yield (1), No. of Clusters (1), Start/End of Milking (1)	9
Other	Month Number (3), Time (2), Cow ID (1), Date (1), Day Length (1), Herd ID (1), Test Day (1), Weekday (1), Year (1)	9
Sensors	Accelerometer (27), Image Data (7), Pedometer (6), Depth Image Data (4), GPS Data (4), Magnetometer Data (3), Gyroscope Data (2), Mass Spectrometry Data (2), RGB Image Data (2), Sound Data (2), 2D Image Data (1), 3D Depth Image Data (1), Audio Data (1), Differential Scanning Calorimetry (DSC) Data (1), Ear Surface Temperature (1), ECG (1), Electromyography (1), Fourier Transformed Infrared Spectroscopy (FTIR) Data (1), Locomotion Score (1), Near Infrared Reflectance (NIR) Spectrophotometer Data (1), NIR Image Data (1), Pressure Sensor (1), Radar (1), RFID Data (1), Spectroscopic Data (1), Thermal Imaging Data (1), Thermo-Hygrometric Sensor Data (1)	27
Soil Characteristics	Soil Boron (1), Soil Calcium (1), Soil Characteristics (1), Soil Copper (1), Soil Iron (1), Soil Magnesium (1), Soil Manganese (1), Soil Organic Matter (1), Soil Ph (1), Soil Phosphorus (1), Soil Potassium (1), Soil Sodium (1), Soil Sulphur (1), Soil Zinc (1)	14

## Appendix C

The specific algorithms used per algorithm category are shown in Table A3, with the number of studies that employed each algorithm presented in brackets next to the algorithm. The total number of features per category is also shown.

**Table A3:** Specific algorithms used per algorithm category.

Algorithm Category	Algorithms (Number of Studies)	n
Bayes	Naïve Bayes (21), Bayes net (5), Gaussian Naïve Bayes (2), Bayes-A (1), Bayesian-LASSO (1), Naïve Bayes updatable (1)	6
Clustering	DBSCAN (1), k-means clustering (1)	2
Meta	Bagging (5), Adaboost (4), Random Subspace (2), rotation forest (2), Boosting (1), Bootstrap Aggregation (1), Super Learner (1), Stacking (1), Voting (1)	9
Neural Network	ANN (46), CNN (10), LSTM (5), Adaptive Neuro-Fuzzy Inference System (2), Faster R-CNN (2), YOLOv2 CNN (2), ANFIS (1), Bi-LSTM (1), CNN Ensemble (1), Extreme Learning Machine (1), Kernel Extreme Learning Machine (1), MLANFIS (1), Mask R-CNN (1), Neuro-Fuzzy Systems (1), Radial Basis Function Network (1), YOLOv3 CNN (1)	16
Other	SVM (31), KNN (20), ANOVA (2), SMO (2), 3-dimensional surface fitting (1), Genetic Algorithm (1), Gaussian Processes (1), Kstar (1), LWL (1), multi-class SVM (1), Multivariate Adaptive Regression Spline (1), one-class SVM (1), Quick Classifier (1)	13
Rule	OneR (3), Jrip (2), PART (2), Classification Based on Associations (1), Majority Voting Rule (1), ZeroR (1)	6
Statistical Regression	Logistic Regression (18), Multiple Linear Regression (13), Linear Discriminant Analysis (6), PLS (6), Linear Regression (4), GAM (3), Multivariate Logistic Regression (3), Ridge Regression (2), Genomic BLUP (1), General Linear Model (1), Logistics (1), MLR with Regularization (1), Multinomial Regression (1), Penalised Linear Regression (1), PLS Discriminant Analysis (1), PLS Regression (1), Simple Logistic (1), Stochastic Gradient Descent (1)	18
Tree	RF (50), DT (26), Gradient Boosting Machine (4), C4.5 (3), CART (3), XGBoost (3), Alternating DT (2), Binary Tree (2), ExtraTrees (2), Gradient Boosted DT (2), J48 (2), M5P Tree (2), Decision Stumps (1), Hoeffding (1), Logistic Model Trees (1), Parallel DT (1), Predictive Clustering Trees (1), Random Tree (1), REPTree (1), Stump DT (1)	20

ANFIS = Adaptive Neuro-Fuzzy Inference System; ANN = Artificial Neural Network; ANOVA = Analysis of variance; CART = Classification and Regression Tree; CNN = Convolutional Neural Network; DBSCAN = Density-Based Spatial Clustering of Applications with Noise; DT = Decision Tree; GAM = Generalised Additive Model; KNN = k-Nearest Neighbor; LSTM = Long Short Term Memory Network; LWL = Locally weighted learning; MLANFIS = Multi-Layered Adaptive Neural Fuzzy Inference System; PART = Projective Adaptive Resonance Theory; PLS = Partial Least Squares; RF = Random Forest; SMO = Sequential Minimal Optimization; SVM = Support Vector Machine.

## Appendix D

The journals that published less than four studies included in this mapping study and all conference proceedings are shown in Table A4, with the number of studies published in each journal/conference presented in brackets next to each journal/conference.

**Table A4:** List of journals that published less than four studies included in this study and all conference proceedings.

Category	Source (Number of Studies)	n
Journals	Applied Animal Behavior Science (3), Biosystems Engineering (3), International Journal of Agricultural and Biological Engineering (2), Irish Veterinary Journal (2), Science Advances (2), African Journal of Science, Technology, Innovation and Development (1), Agricultural Systems (1), Agronomy (1), Animal (1), Applied Energy (1), Applied Sciences (1), Archives Animal Breeding (1), BMC Veterinary Research (1), BioData Mining (1), Ciencia Rural (1), Computational and Mathematical Methods in Medicine (1), Food Control (1), Genetics Selection Evolution (1), Genetics and Molecular Research (1), IEEE Geoscience and Remote Sensing Letters (1), Information Processing in Agriculture (1), Journal of Energy Technology and Policy (1), Journal of Food Composition and Analysis (1), Journal of Systems Architecture (1), Livestock Science (1), Multimodal Technologies and Interaction (1), Research in Veterinary Science (1), Theriogenology (1)	28
Conferences	IEEE Sensors (2), International Conference on Unmanned Systems and Artificial Intelligence (ICUSAI) (2), ABASE Annual International Meeting (1), Africa Week Conference (IST) (1), Consumer Communications and Networking Conference (CCNC) (1), European Conference on Electrical Engineering and Computer Science (EECS) (1), International Conference on Big Data Computing Service and Applications (1), International Conference on Biometrics Theory, Applications and Systems (BTAS) (1), International Conference on Computers and Their Applications (CATA) (1), International Conference on Computing for Sustainable Global Development (INDIACom) (1), International Conference on Data Mining Workshops (1), International Conference on Data and Software Engineering (ICoDSE) (1), International Conference on Intelligent Robots and Systems (IROS) (1), International Electronics Symposium (IES) (1), International Seminar on Application for Technology of Information and Communication (iSemantic) (1), International Symposium on Personal, Indoor and Mobile Radio Communications (PIMRC) (1), International conference on Bio Signals, Images, and Instrumentation (ICBSII) (1), Journal of Physics: Conference Series (1)	18

## Appendix E

The specific feature data, dependent variables, machine learning algorithms, evaluation metrics and evaluation methods used per research category for each of the 129 publications included in this mapping study are shown in Table A5.

**Table A5:** Feature data, dependent variables, algorithms, evaluation metrics and methods used per study.

Animal Husbandry					
Study	Features	Dependent	Algorithms ^a^	Evaluation Metrics ^b^	Evaluation Methods ^c^
[14]	Calving/Pregnancy Information, Cow Characteristics and Clinical Information, Milk Characteristics, Sensor Data	Calving Difficulty	multinomial regression, DT, RF, ANN	Recall, Specificity, F_1_ Score, Accuracy	Hold-Out
[15]	Calving/Pregnancy Information, Cow Characteristics and Clinical Information, Lactation Information, Milk Characteristics	Submission Rate	DT, KNN, RF, ANN, LR	Accuracy, Balanced Accuracy, Recall, Specificity, PPV, NPV, F_1_ Score, Cohen’s Kappa, Prevalence, AUC, MAE	Repeated k-fold CV, Hold-Out
[5]	Calving/Pregnancy Information, Cow Characteristics and Clinical Information, Farm Characteristics and Management	First-Service Conception Rate	Alternating DT, LR	Accuracy, FP, FN	k-fold CV
[5]	Calving/Pregnancy Information, Cow Characteristics and Clinical Information, Farm Characteristics and Management, Milk Characteristics	Pregnancy Status	Alternating DT, LR	Accuracy, FP, FN	k-fold CV
[16]	Diet/Feeding	Estrus Detection	GLM, ANN, RF	Accuracy, Recall, Specificity, PPV, NPV, Error Rate	Nested CV
[17]	Calving/Pregnancy Information, Cow Characteristics and Clinical Information, Milk Characteristics	Pregnancy Status	DT	Accuracy, Recall, Specificity, PPV, NPV	Hold-Out
[18]	Cow Characteristics and Clinical Information	Cow Survival	Naïve Bayes, RF, LR	Accuracy, Recall, Specificity, AUC	k-fold CV, Hold-Out
[19]	Cow Characteristics and Clinical Information, Milk Characteristics	Genomic Evaluation	Random-Boosting, Genomic BLUP, Bayesian-LASSO, Bayes-A	MSE, r	Hold-Out
[20]	Cow Characteristics and Clinical Information, Milk Characteristics, Milking Parameters	Estrus Detection	DT, Naïve Bayes, SVM, RF, LR	Accuracy, PPV, Recall, F_1_ Score, Specificity	Train/Validation/Test
[21]	Calving/Pregnancy Information, Cow Characteristics and Clinical Information	Conception Performance	ANN, multivariate adaptive regression spline, LR	RMSE, AIC, AUC, Bayesian Information Criterion, Generalized Cross-Validation Error, Accuracy	k-fold CV, Hold-Out
[22]	Sensor Data	Calving Prediction	LSTM, Bi-LSTM	Recall, Specificity, PPV, NPV	Hold-Out
[23]	Calving/Pregnancy Information	Estrus Detection	Multivariate LR	Accuracy	Hold-Out
[24]	Sensor Data	Estrus Detection	Pre-trained	Recall, Specificity, PPV, NPV, Accuracy, Error Rate	Hold-Out
[25]	Sensor Data	Estrus Detection	K-means clustering	n/a	Hold-Out
[26]	Cow Characteristics and Clinical Information	Cow Survival	majority voting rule, multivariate LR, RF, Naïve Bayes	PPV, Recall, Balanced Accuracy, AUC	Repeated k-fold CV, Hold-Out
[27]	Calving/Pregnancy Information, Cow Characteristics and Clinical Information, Lactation Information, Milk Characteristics, Other	Conception Success	C4.5 DT, Naïve Bayes, Bayesian network, LR, SVM, PLS, RF, rotation forest	AUC	Repeated k-fold CV
[28]	Calving/Pregnancy Information, Cow Characteristics and Clinical Information, Lactation Information, Milk Characteristics, Other	Abortion Incidence	Naïve Bayes, Bayesian network, DT, RF, OneR, PART, LR, ANN, stochastic gradient descent, bagging, boosting, rotation forest	F_1_ Score, AUC, PPV, MCC, Recall, Lift	Hold-Out
[29]	Sensor Data	Estrus Detection	KNN, ANN, LDA, DT	Recall, Specificity, PPV, NPV, Accuracy, F_1_ Score	k-fold CV
[30]	Sensor Data	Calving Prediction	RF, LDA, ANN	Accuracy, Recall, Specificity	LOOCV, Hold-Out
[31]	Diet/Feeding, Farm Characteristics and Management	Conception rate	M5P Tree, ANOVA	r, RMSE	k-fold CV
[31]	Diet/Feeding, Farm Characteristics and Management	Service Rates	M5P Tree, ANOVA	r, RMSE	k-fold CV
[32]	Sensor Data	Estrus Detection	LSTM, CNN, KNN	Recall, Specificity, PPV	Hold-Out
[33]	Milk Characteristics	Pregnancy Status	PLS discriminant analysis, CNN	PPV, Recall, F_1_ Score	k-fold CV
[34]	Calving/Pregnancy Information, Cow Characteristics and Clinical Information, Milk Characteristics	Pregnancy Status	Naïve Bayes, Bayesian networks, DT, DT ensemble, RF	AUC, FP, TP	k-fold CV
[35]	Sensor Data	Pregnancy Status	not specified	Recall, Specificity	Hold-Out
[36]	Calving/Pregnancy Information, Cow Characteristics and Clinical Information, Lactation Information, Milk Characteristics	Pregnancy Status	GAM, LR, bagging	PPV, Recall, F_1_ Score, AUC	Hold-Out
[37]	Calving/Pregnancy Information, Cow Characteristics and Clinical Information, Milk Characteristics, Other	Conception Probability	GAM, LR	Recall, Specificity, Accuracy, PPV, NPV, AUC, MCC	Hold-Out
[38]	Sensor Data	Calving Prediction	RF	MCC, AUC, Recall, Specificity	Hold-Out
**Behavior Analysis**					
**Study**	**Features**	**Dependent**	**Algorithms**	**Evaluation Metrics**	**Evaluation Methods**
[39]	Sensor Data	Cow Activity	RF, Naïve Bayes, Jrip, J48	Accuracy, FP, F_1_ Score, AUC	Repeated k-fold CV
[40]	Sensor Data	Cow Activity	RF	Accuracy	k-fold CV
[41]	Diet/Feeding, Sensor Data	Jaw Movements	DT, RF, ANN, radial basis function network, SVM, extreme learning machine	Accuracy, Recall, PPV	LOOCV
[42]	Sensor Data	Cow Detection	YOLOv2 CNN	Accuracy	Hold-Out
[43]	Sensor Data	Cow Activity	KNN, SVM, ANN	Accuracy, PPV, Recall, Specificity, F_1_ Score, Cohen’s Kappa	LOOA
[44]	Sensor Data	Cow Activity	SVM, Naïve Bayes, KNN, RF, LR	F_1_ Score, Recall, PPV	Nested CV
[45]	Cow Characteristics and Clinical Information, Sensor Data	Cow Activity	RF, LDA, ANN	Recall, Specificity, Accuracy	k-fold CV, Hold-Out
[46]	Sensor Data	Cow Activity	Bagging, Random Subspace, AdaBoost, Binary Tree, LDA classifier, Naïve Bayes, KNN, Adaptive Neuro-Fuzzy Inference System	Accuracy, Recall, Specificity, F_1_ Score, FDR	Hold-Out
[47]	Sensor Data	Cow Activity	DT, SVM	PPV, Recall, Specificity	Nested CV
[48]	Cow Characteristics and Clinical Information, Sensor Data	Cow Activity	SVM, DT	Accuracy	Hold-Out
[49]	Sensor Data	Cow Detection	ANN, KNN	PPV, Recall, F_1_ Score, Accuracy, Hamming loss	Hold-Out
[50]	Sensor Data	Cow Activity	DT, ANN	Accuracy, Recall, Specificity	k-fold CV, Train/Validation/Test
[51]	Sensor Data	Cow Activity	Extreme Boosting Algorithm, SVM, Adaboost, RF	Accuracy, Cohen’s Kappa, Recall, Specificity	Repeated k-fold CV
[52]	Sensor Data	Cow Activity	Bagging, Random Subspace, AdaBoost, Binary Tree, LDA, Naïve Bayes, KNN, Adaptive Neuro-Fuzzy Inference System	Accuracy, Recall, Specificity, F_1_ Score, FDR	Hold-Out
[53]	Sensor Data	Cow Detection	Faster Region CNN, k-means clustering, DBSCAN	n/a	n/a
[54]	Sensor Data	Cow Identification	Mask R-CNN	TP, FP, FN, IoU, PPV, Recall, Averaged PPV, mAP, AR	Hold-Out
[55]	Sensor Data	Cow Activity	KNN	PPV, Recall	Repeated Hold-Out
[56]	Sensor Data	Cow Activity	Adaboost	Accuracy, Specificity, Recall, PPV, F_1_ Score, Cohen’s Kappa	k-fold CV
[57]	Cow Characteristics and Clinical Information, Sensor Data	Sleep Stages	ANN, RF	AUC, Accuracy, F_1_ Score, PPV, Recall	k-fold CV
[58]	Cow Characteristics and Clinical Information, Sensor Data	Cow Udder Anomalies	KNN, ANN, LSTM, DT	Recall, FPR	Repeated Train/Validation/Test
[59]	Sensor Data	Cow Activity	KNN, Naïve Bayes, SVM	PPV, Recall, Accuracy	LOOA
[60]	Sensor Data	Cow Activity	CNN, LSTM	Accuracy	Train/Validation/Test
[61]	Sensor Data	Cow Identification	KNN, SVM, RF, DT, LR	Accuracy	Hold-Out
[62]	Sensor Data	Cow Activity	Naïve Bayes, Bayes net, SVM, ANN, Jrip, PART, OneR, Naïve Bayes, J48, logistic model trees, meta (super learner), LR, Simple Logistic	Accuracy, Recall, Specificity, PPV, F_1_ Score, Training Speed	k-fold CV
**Feeding**					
**Title**	**Features**	**Dependent**	**Algorithms**	**Evaluation Metrics**	**Evaluation Methods**
[63]	Calving/Pregnancy Information, Cow Characteristics and Clinical Information, Diet/Feeding, Lactation Information, Meteorological Conditions, Milking Parameters	Concentrate Feed Intake	ANN	MSE	Hold-Out
[64]	Milk Characteristics	Volatile Fatty Acids	ANN, MLR	MSPE, RMSE, RMSE %	Train/Validation/Test
[65]	Sensor Data	Insufficient Herbage Allowance	SVM, RF, XGBoost	AUC, Recall, Specificity, Accuracy, PPV, F_1_ Score	LOOA
[66]	Cow Characteristics and Clinical Information, Lactation Information, Milk Characteristics	Dry Matter Intake	ANN, PLS	CCC, RMSE, Mean Bias, R^2^	k-fold CV, Hold-Out
[67]	Calving/Pregnancy Information, Cow Characteristics and Clinical Information, Lactation Information, Milk Characteristics	Dry Matter Intake	ANN, PLS	R^2^, RMSE, RPD	Repeated k-fold CV
[68]	Diet/Feeding	Diet Energy Digestion	kernel extreme learning machine, Linear Regression, ANN, SVM, Extreme Learning Machine	MAE, MAPE, RMSE, R^2^, Training Speed	k-fold CV, Repeated Hold-Out
[69]	Sensor Data	Feeding Behavior	CNN	Accuracy	Hold-Out
[70]	Cow Characteristics and Clinical Information, Diet/Feeding, Milk Characteristics	Residual Feed Intake	SVM	MSE, r	Repeated Hold-Out
**Management**					
**Title**	**Features**	**Dependent**	**Algorithms**	**Evaluation Metrics**	**Evaluation Methods**
[71]	Cow Characteristics and Clinical Information	Methane Emissions	Ridge Regression, RF	R^2^	Repeated k-fold CV
[72]	Farm Characteristics and Management, Milk Characteristics	Electricity use	SVM	RPE, CCC, MAPE, MAE, MPE, r, RMSE	Hold-Out
[73]	Farm Characteristics and Management	Energy Output	ANN	R^2^, RMSE, MAPE	Train/Validation/Test
[74]	Farm Characteristics and Management, Meteorological Conditions, Milk Characteristics, Milking Parameters	Electricity use	MLR, SVM	RPE, CCC, MPE, RMSE	Hold-Out
[75]	Calving/Pregnancy Information, Farm Characteristics and Management	Electricity use	MLR	RPE, R^2^	LOOCV
[75]	Calving/Pregnancy Information, Farm Characteristics and Management	Diesel use	MLR	RPE, R^2^	LOOCV
[76]	Farm Characteristics and Management, Meteorological Conditions, Milk Characteristics, Milking Parameters	Electricity use	ANN, RF, DT, SVM, MLR	RMSE, RPE, CCC, MSPE, MPE, r	Nested CV
[76]	Farm Characteristics and Management, Meteorological Conditions, Milk Characteristics	Water use	ANN, RF, DT, SVM, MLR	RMSE, RPE, CCC, MSPE, MPE, r	Nested CV
[77]	Farm Characteristics and Management	Energy Output	MLANFIS	R^2^, RMSE, MAPE	Train/Validation/Test
[78]	Farm Characteristics and Management	Energy Output	ANFIS	R^2^, RMSE, MAPE	Train/Validation/Test
[79]	Farm Characteristics and Management, Meteorological Conditions, Milk Characteristics	Electricity use	MLR	R^2^	Hold-Out
[80]	Farm Characteristics and Management, Meteorological Conditions, Milk Characteristics, Milking Parameters	Electricity use	MLR	RMSE, RPE, CCC, MSPE, MPE, r	k-fold CV
[80]	Farm Characteristics and Management, Meteorological Conditions, Milk Characteristics	Water use	MLR	RMSE, RPE, CCC, MSPE, MPE, r	k-fold CV
[81]	Diet/Feeding	Faeces Output	SVM, ANN, LR	RMSE, norm-RMSE	Repeated k-fold
[81]	Diet/Feeding	Urine Output	SVM, ANN, LR	RMSE, norm-RMSE	Repeated k-fold
[81]	Diet/Feeding	Faecal Nitrogen	SVM, ANN, LR	RMSE, norm-RMSE	Repeated k-fold
[81]	Diet/Feeding	Urinary Nitrogen	SVM, ANN, LR	RMSE, norm-RMSE	Repeated k-fold
[82]	Meteorological Conditions, Other	Methane Emissions	SVM, RF, ensemble, gradient boosting, ridge regression, ANN, gaussian processes, MLR with regularization, MLR	RMSE, R^2^, MAE	Nested CV
[83]	Farm Characteristics and Management, Meteorological Conditions, Other	Manure Temperature	gradient boosted trees, bagged tree ensembles, RF, ANN	MAE, RMSE, R^2^	Train/Validation/Test
[84]	Diet/Feeding, Farm Characteristics and Management, Meteorological Conditions, Soil Characteristics1	Herbage Production	predictive clustering trees, RF	R^2^, RRMSE	k-fold CV
[84]	Diet/Feeding, Farm Characteristics and Management, Meteorological Conditions, Soil Characteristics1	Nutrient Concentration	predictive clustering trees, RF	R^2^, RRMSE	k-fold CV
**Milk**					
**Study**	**Features**	**Dependent**	**Algorithms**	**Evaluation Metrics**	**Evaluation Methods**
[85]	Farm Characteristics and Management, Milk Characteristics, Milking Parameters, Other	Milk Bacterial Index	C4.5, REPTree, RF, Random Tree, Hoeffding, Decision Stumps, ANN, SVM, Logistics, SMO, LWL, Kstar, KNN, Naïve Bayes, Naïve Bayes updateable, OneR, ZeroR, Adaboost, Bagging, Stacking, Voting	MAPE	Hold-Out
[86]	Cow Characteristics and Clinical Information, Meteorological Conditions	Milk Production	ANN	MSE	Train/Validation/Test
[63]	Calving/Pregnancy Information, Cow Characteristics and Clinical Information, Diet/Feeding, Lactation Information, Meteorological Conditions, Milking Parameters	Milk Parameters	ANN	MSE	Hold-Out
[87]	Diet/Feeding, Farm Characteristics and Management, Soil Characteristics1	Milk Production	CART	n/a	Tree Analysis
[88]	Calving/Pregnancy Information, Diet/Feeding, Lactation Information, Milking Parameters	Milk Production	SVM, ANN, RF, MLR	RMSE, MAE, R^2^	k-fold CV
[89]	Calving/Pregnancy Information, Lactation Information, Milk Characteristics, Milking Parameters	Milk Quality Parameters	GAM, RF, ANN	MSE	k-fold CV
[90]	Sensor Data	Milk Adulteration	DT, Naïve Bayes, LDA, SVM, ANN	Accuracy, Recall, Specificity, FP, FN, FPR, AUC	Train/Validation/Test
[91]	Sensor Data	Milk Adulteration	RF, gradient boosting machine, ANN	Accuracy, Specificity, Recall	Hold-Out
[92]	Milk Characteristics	Milk Production	DT, ANN	Accuracy	k-fold CV, Hold-Out
[93]	Calving/Pregnancy Information, Cow Characteristics and Clinical Information, Lactation Information, Milk Characteristics	Outlier Lactations	CART	Recall, Specificity, TP, FP, PPV	k-fold CV
[94]	Milk Characteristics	Milk Metabolites	RF, PLS	r	k-fold CV
[95]	Milk Characteristics	Milk Adulteration	ANN	r	Train/Validation/Test
[96]	Sensor Data	Milk Quality Parameters	ANN, PLS	MSE	Train/Validation/Test
[97]	Sensor Data	Milk Adulteration	CNN, RF, Gradient Boosting Machine, LR, Linear Regression, PLS	Accuracy, AUC	Hold-Out
[98]	Cow Characteristics and Clinical Information, Lactation Information, Other	Milk Production	RF, ANN, MLR	CCC, r	k-fold CV, Hold-Out
[99]	Cow Characteristics and Clinical Information, Lactation Information, Meteorological Conditions, Milk Characteristics, Other	Fat EBV	ANN, neuro-fuzzy systems	RMSE, r	Train/Validation/Test
[99]	Cow Characteristics and Clinical Information, Lactation Information, Meteorological Conditions, Milk Characteristics, Other	Milk EBV	ANN, neuro-fuzzy systems	RMSE, r	Train/Validation/Test
[100]	Calving/Pregnancy Information, Farm Characteristics and Management, Lactation Information, Meteorological Conditions, Milk Characteristics, Sensor Data	Milk Production	RF	RPE	k-fold CV, Hold-Out
**Physiology and Health**					
**Study**	**Features**	**Dependent**	**Algorithms**	**Evaluation Metrics**	**Evaluation Methods**
[101]	Sensor Data	Animal Dimensions	MLR	R^2^, RMSE, MRAE	Hold-Out
[71]	Cow Characteristics and Clinical Information	Milk Productivity	Ridge Regression, RF	R^2^	Repeated k-fold CV
[71]	Cow Characteristics and Clinical Information	Rumen and Blood Metabolites	Ridge Regression, RF	R^2^	Repeated k-fold CV
[102]	Calving/Pregnancy Information, Cow Characteristics and Clinical Information, Farm Characteristics and Management, Lactation Information, Milk Characteristics	Lameness Detection	CART, gradient boosted machine, extreme gradient boosting, RF, Multivariate LR	AUC, Recall, Specificity	Repeated k-fold CV
[103]	Sensor Data	Lameness Detection	one-class SVM	Accuracy, Specificity, Recall	LOOCV
[104]	Sensor Data	Body Condition Score	CNN, YOLO-v3 CNN	IoU, Mean IoU, Accuracy, PPV, fps, Model Size	Hold-Out
[105]	Sensor Data	Lameness Detection	SVM, KNN	Accuracy, TN, TP, FN, FP	Repeated Hold-Out
[106]	Calving/Pregnancy Information, Cow Characteristics and Clinical Information, Lactation Information, Milk Characteristics	Mastitis Detection	RF	Accuracy, Recall, Specificity, F_1_ Score, Cohen’s Kappa, PPV, NPV	Repeated k-fold CV, Hold-Out
[107]	Sensor Data	Body Condition Score	DT, ANN, Linear Regression	MAE, R^2^	k-fold CV
[108]	Sensor Data	Body Condition Score	3-dimensional surface fitting	MAE, MBE, R^2^	Hold-Out
[109]	Sensor Data	Body Condition Score	CNN	Accuracy, PPV, Recall, F_1_ Score	Hold-Out
[110]	Cow Characteristics and Clinical Information, Diet/Feeding, Farm Characteristics and Management, Meteorological Conditions, Milk Characteristics	Heat Stress	DT	Accuracy	Hold-Out
[111]	Calving/Pregnancy Information, Cow Characteristics and Clinical Information, Milk Characteristics, Sensor Data	Ketosis Detection	Naïve Bayes	Accuracy, Recall, Specificity, PPV, Youdens Index, Cohen’s Kappa, MCC, NPV	k-fold CV
[112]	Sensor Data	Body Condition Score	Faster R-CNN	IoU, TP, TN, FP, FN, Accuracy, PPV, Average PPV, Average PPV, fps	Hold-Out
[113]	Cow Characteristics and Clinical Information	Mastitis Detection	SVM, RF, Naïve Bayes, ANN	Accuracy, AUC	Nested CV
[114]	Sensor Data	Body Condition Score	CNN (pre-trained)	Accuracy, Training Speed, Model Size	Hold-Out
[115]	Calving/Pregnancy Information, Cow Characteristics and Clinical Information, Lactation Information, Milk Characteristics	Lameness Detection	ANN	Accuracy	Hold-Out
[116]	Sensor Data	Mastitis Detection	GA, Supervised ANN, quick classifier	Cohen’s Kappa, Recall, Specificity, PPV, NPV, Accuracy	Repeated Hold-Out
[117]	Sensor Data	Lameness Detection	multi-class SVM	Accuracy, PPV	k-fold CV
[118]	Sensor Data	Body Condition Score	CNN, ensemble	Accuracy, PPV, Recall, F_1_ Score,	Hold-Out
[119]	Calving/Pregnancy Information, Cow Characteristics and Clinical Information, Lactation Information, Milk Characteristics	Bodyweight	RF	r, CCC, R^2^, RMSE, MAE, RPD, RPIQ	Repeated k-fold CV
[120]	Milk Characteristics, Milking Parameters	Mastitis Detection	DT, Stump DT, Parallel DT, RF	Accuracy, Info Gain, Gini Index, Gain Ratio	k-fold CV
[121]	Sensor Data	Digital Dermatitis	YOLOv2 architecture	Accuracy, Cohen’s Kappa	Hold-Out
[122]	Calving/Pregnancy Information, Cow Characteristics and Clinical Information, Lactation Information, Milk Characteristics	Mastitis Detection	M5P Tree, ANOVA	Accuracy	Train/Validation/Test
[123]	Sensor Data	Lameness Detection	SVM, RF, KNN, DT	Accuracy	Hold-Out
[124]	Cow Characteristics and Clinical Information, Farm Characteristics and Management, Milk Characteristics, Milking Parameters	Mastitis Detection	C4.5	Accuracy	Repeated k-fold CV
[125]	Sensor Data	Lameness Detection	RF, KNN, SVM, DT	Accuracy	Hold-Out
[126]	Milk Characteristics	Metabolic Status	SMO, RF, alternating DT, Naïve Bayes Updatable	Accuracy, Recall, Specificity, PPV, F_1_ Score	LOOA
[127]	Cow Characteristics and Clinical Information, Lactation Information, Meteorological Conditions, Milk Characteristics	Heat Stress	DT, MLR	Recall, Specificity, Balanced Accuracy, Accuracy	Hold-Out
[128]	Cow Characteristics and Clinical Information, Lactation Information	Mastitis Detection	DT, RF, Naïve Bayes	Accuracy, Recall, Specificity, AUC	k-fold CV, Hold-Out
[129]	Milk Characteristics	Tuberculosis Status	CNN	Accuracy, Specificity, PPV, NPV, Recall, MCC	Hold-Out
[130]	Sensor Data	Mastitis Detection	SVM, RF, ANN, Adaboost, Naïve Bayes, LR	Recall, Specificity, Accuracy, Cohen’s Kappa	Nested CV
[131]	Calving/Pregnancy Information, Cow Characteristics and Clinical Information, Lactation Information, Milk Characteristics, Sensor Data	Lameness Detection	Gradient Boosted DT	Accuracy, AUC, Recall, Specificity	k-fold CV, Hold-Out
[132]	Calving/Pregnancy Information, Cow Characteristics and Clinical Information, Lactation Information, Milk Characteristics	Metabolic Status	DT, Naïve Bayes, Bayesian Network, SVM, ANN, Bootstrap Aggregation, RF, KNN	PPV, NPV, Recall, Specificity, Error Rate	Repeated k-fold CV
[133]	Meteorological Conditions	Respiration Rate	penalized linear regression, RF, gradient boosted machines, ANN	RMSE, MAE, R^2^	Train/Validation/Test
[133]	Meteorological Conditions	Skin Temperature	penalized linear regression, RF, gradient boosted machines, ANN	RMSE, MAE, R^2^	Train/Validation/Test
[133]	Meteorological Conditions	Vaginal Temperature	penalized linear regression, RF, gradient boosted machines, ANN	RMSE, MAE, R^2^	Train/Validation/Test
[134]	Sensor Data	Teat Cleanliness	KNN	Cohen’s Kappa	k-fold CV, Hold-Out
[135]	Milk Characteristics, Milking Parameters	Mastitis Detection	classification based on associations	Accuracy, Recall, Specificity, F_1_ Score, PPV, AUC	Repeated k-fold CV
[136]	Calving/Pregnancy Information, Cow Characteristics and Clinical Information, Diet/Feeding, Lactation Information, Milk Characteristics, Milking Parameters, Sensor Data	Mastitis Detection	RF, Gaussian Naïve Bayes, ExtraTrees, LR	PPV, AUC, Recall, Specificity	Repeated k-fold CV
[136]	Calving/Pregnancy Information, Cow Characteristics and Clinical Information, Diet/Feeding, Lactation Information, Milk Characteristics, Milking Parameters, Sensor Data	Lameness Detection	RF, Gaussian Naïve Bayes, ExtraTrees, LR	PPV, AUC, Recall, Specificity	Repeated k-fold CV
[137]	Meteorological Conditions, Sensor Data	Heat Stress	ANN, Linear Regression	Mean Error, RMSE, R^2^	Train/Validation/Test
[138]	Cow Characteristics and Clinical Information, Diet/Feeding, Sensor Data	Noxious Events	RF, SVM, DT, KNN, Naïve Bayes	PPV, NPV, Accuracy	Hold-Out
[139]	Sensor Data	Heat Stress	LSTM	MAE, RMSE	Train/Validation/Test
[140]	Calving/Pregnancy Information, Cow Characteristics and Clinical Information, Diet/Feeding, Lactation Information, Meteorological Conditions, Milk Characteristics, Milking Parameters, Other, Sensor Data	Mastitis Detection	RF, SVM, KNN, Gaussian Naïve Bayes, Extra Trees Classifier, LR	AUC, Recall, Specificity, Accuracy, PPV, F_1_ Score	Hold-Out
[140]	Calving/Pregnancy Information, Cow Characteristics and Clinical Information, Diet/Feeding, Lactation Information, Meteorological Conditions, Milk Characteristics, Milking Parameters, Other, Sensor Data	Lameness Detection	RF, SVM, KNN, Gaussian Naïve Bayes, Extra Trees Classifier, LR	AUC, Recall, Specificity, Accuracy, PPV, F_1_ Score	Hold-Out
[141]	Calving/Pregnancy Information, Lactation Information, Milk Characteristics	Bodyweight	PLS Regression	RMSE	k-fold CV, Hold-Out

^a^ Description of algorithm abbreviations can be found in Appendix D. ^b^ AR = Averaged Recall Score; AUC = area under the ROC curve; CCC = concordance correlation coefficient; FDR = False Discovery Rate; FPR = False Positive Rate; FN = false negative; FP = false positive; fps = Frame per Second; IoU = Intersection over Union; mAP = Averaged Precision Score; MAE = mean absolute error; MAPE = mean absolute percentage error; MBE = Mean Bias Error; MCC = Matthew’s Correlation Coefficient; MPE = mean percentage error; MSE = mean square error; MSPE = mean square percentage error; NPV = negative predictive value; PPV = positive predictive value; R^2^ = coefficient of determination; RMSE = root mean squared error; RPE = relative prediction error; RPD = the ratio of performance to deviation; RPIQ = the ratio of performance to the interquartile range; TN = True Negative; TP = True Positive. ^c^ LOOA = leave-out-one-animal; LOOCV = leave-one-out cross-validation; Nested CV = nested cross-validation; k-fold CV = k-fold cross-validation.
